# Crocins, the Bioactive Components of *Crocus sativus* L., Counteract the Disrupting Effects of Anesthetic Ketamine on Memory in Rats

**DOI:** 10.3390/molecules26030528

**Published:** 2021-01-20

**Authors:** Nikolaos Pitsikas, Petros A. Tarantilis

**Affiliations:** 1 Department of Pharmacology, Faculty of Medicine, School of Health Sciences, University of Thessaly, Biopolis, Panepistimiou 3, 415-00 Larissa, Greece; 2 Laboratory of Chemistry, Department of Food Science and Human Nutrition, School of Food and Nutritional Sciences, Agricultural University of Athens, 115-27 Athens, Greece; ptara@aua.gr

**Keywords:** crocins, anesthetic ketamine, memory, rat

## Abstract

Consistent experimental evidence suggests that anesthetic doses of the non-competitive *N*-methyl-d-aspartate (NMDA) receptor antagonist ketamine cause severe memory impairments in rodents. Crocins are among the various bioactive ingredients of the plant *Crocus sativus* L., and their implication in memory is well-documented. It has not yet been elucidated if crocins are able to attenuate the memory deficits produced by anesthetic ketamine. The present study was undertaken aiming to clarify this issue in the rat. For this aim, the object recognition, the object location and the habituation tests, reflecting non-spatial recognition memory, spatial recognition memory and associative memory, respectively, were utilized. A post-training challenge with crocins (15–30 mg/kg, intraperitoneally (i.p.), acutely) counteracted anesthetic ketamine (100 mg/kg, i.p.)-induced performance impairments in all the above-mentioned behavioral memory paradigms. The current findings suggest that crocins modulate anesthetic ketamine’s amnestic effects.

## 1. Introduction

Ketamine is a drug largely utilized in clinical and veterinary medicine due to its important anesthetic and analgesic properties [1,2]. Ketamine binds to the phencyclidine (PCP) binding site within the pore of the channel of the *N*-methyl-d-aspartate (NMDA) receptor and exerts its effects as a non-competitive antagonist [3]. Exposure to a low-dose range of ketamine (sub-anesthetic doses) induces schizophrenia-like symptoms, including memory impairments, both in humans and rodents [2].

By contrast, anesthetic doses of ketamine cause an anesthetic state called “dissociative anesthesia” characterized by severe sensory loss and analgesia, and does not depress the cardiovascular or the respiratory system, but disrupts cognition [4,5]. Regarding the latter, it has been demonstrated that a challenge with anesthetic ketamine disrupted rodents’ anterograde memory [6], memory consolidation [7] and recall of previous information [8,9]. Based on the complexity of the behavioral paradigm used and in agreement with clinical findings [10], 72 h are required for the recovery of memory in rats that receive anesthetic doses of ketamine [6]. Anesthetic ketamine’s adverse behavioral effects are ascribed to its inhibitory action on the NMDA receptor [11] and on the extracellular signal-regulated kinase (ERK) signal transduction pathway [12]. Additionally, anesthetic ketamine promotes the overexpression of the transcriptional marker c-fos [13] and oxidative stress [14].

*Crocus sativus* L. is a plant cultivated in many countries all around the world including Iran, India, Italy, Spain and Greece. The spice saffron is its product. Saffron is the dried red stigmas of the flower. The major components of saffron are crocins, picrocrocin and safranal. [15,16].

The stigmas of *C. sativus* L. are used in traditional medicine as an anti-catarrhal, eupeptic, expectorant and emmenagogue [17]. Modern pharmacological studies have demonstrated that saffron’s crude extracts and purified chemicals possess anti-tumor and anti-inflammatory properties, counteract atherosclerosis and hepatic damage [17], and exert a beneficial action in different pathologies of the central nervous system including depression, schizophrenia and anxiety [18].

In line with the above, a conspicuous number of preclinical and clinical studies propose the involvement of crocins in cognition [19]. Crocins, glycosyl esters of crocetin, are water-soluble carotenoids. It has recently been shown that crocins, despite their highly hydrophilic profile, are capable of penetrating the blood–brain barrier and reaching the central nervous system following intraperitoneal administration in mice [20]. In this context, it has been demonstrated that crocins attenuated the schizophrenia-like behavioral deficits, including cognitive impairments, induced by sub-anesthetic doses of ketamine in rats [21]. In particular, crocins counteracted the disruption of non-spatial recognition memory caused by sub-anesthetic ketamine in a procedure reflecting the modulation of post-training memory components (the storage and/or retrieval of information) [21]. Several lines of evidence suggest that the beneficial effects exerted by crocins on memory might be related to their strong anti-oxidant properties [22,23].

Little is known at present regarding if and how crocins are able to attenuate the cognitive deficits caused by the administration of anesthetic doses of ketamine in animals. In spite of its well-documented amnestic effects, anesthetic ketamine increases cerebral blood flow and metabolism in spontaneously breathing patients and does not influence intracranial pressure [24], and it did not induce neurodegeneration in the developing brain [25]. Due to these interesting properties, ketamine is the anesthetic of choice in specific situations such as patients with compromised hemodynamic profiles or suffering from asthma [24] and in pediatrics [25]. Thus, compounds that might be able to attenuate the cognition problems associated with the administration of anesthetic ketamine might be of high utility in clinical practice.

Furthermore, it has not yet been elucidated if crocins can attenuate the cognitive deficits induced by anesthetic doses of ketamine related to types of memory other than non-spatial recognition memory (e.g., spatial recognition memory and associative memory) and modulate mnemonic components other than the storage and retrieval of information (e.g., the acquisition of information).

Taking the above into account, the aim of our research was to test the efficiency of crocins in attenuating the usual memory problems expressed by rats that have received anesthetic ketamine. For these studies, the object recognition task (ORT), the object location task (OLT) and the habituation test (HT) were used. The ORT and OLT measure non-spatial [26] and spatial recognition memory [27], respectively, while the HT is a non-associative learning task [28].

## 2. Results

In line with prior results, the righting reflex was lost in rats within 8 min, and anesthesia was extinguished in all groups of rats that received ketamine within 30 min, independently of the pharmacological treatment [6,9,29,30].

### 2.1. Experiment 1: Effects of Acute Challenge with Crocins and Anesthetic Ketamine on Animals’ Performance in the ORT

**Figure 1:**
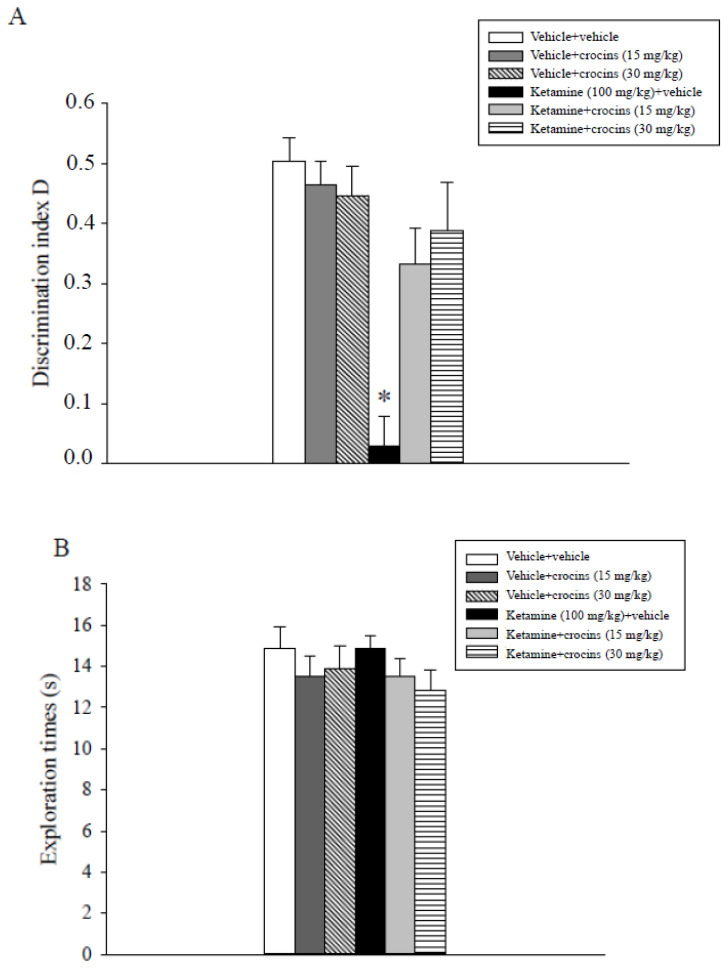
Object recognition task. The histogram represents the mean ± S.E.M. of 8 rats per treatment group. (**A**) Discrimination index D performance expressed by various groups of rats during the choice phase (T2). * *p* < 0.05 vs. all the other groups including the ketamine + crocins (15 mg/kg) and ketamine + crocins (30 mg/kg) groups. (**B**) Total exploration times.

The evaluation of the discrimination index D data evidenced a statistically significant main effect of ketamine (F(1,47) = 25.2, *p* < 0.001), a statistically significant main effect of crocins (F(2,47) = 4.6, *p* = 0.016) and a significant ketamine x crocins interaction (F(2,47) = 8.4, *p* < 0.001). Post hoc comparisons showed that the ketamine + vehicle group displayed an inferior index D with respect to all the other experimental groups, including the ketamine + 15 mg/kg crocins and ketamine + 30 mg/kg crocins groups (*p* < 0.05; Figure 1A).

Assessment of the exploratory activity data obtained for the various groups of rats during the choice phase (T2) did not show significant effects of ketamine, crocins or their combination (Figure 1B). The results indicate that treatment with crocins alleviated the disruption of non-spatial recognition memory caused by anesthetic ketamine.

### 2.2. Experiment 2: Effects of Acute Challenge with Crocins and Anesthetic Ketamine on Animals’ Performance in the OLT

**Figure 2:**
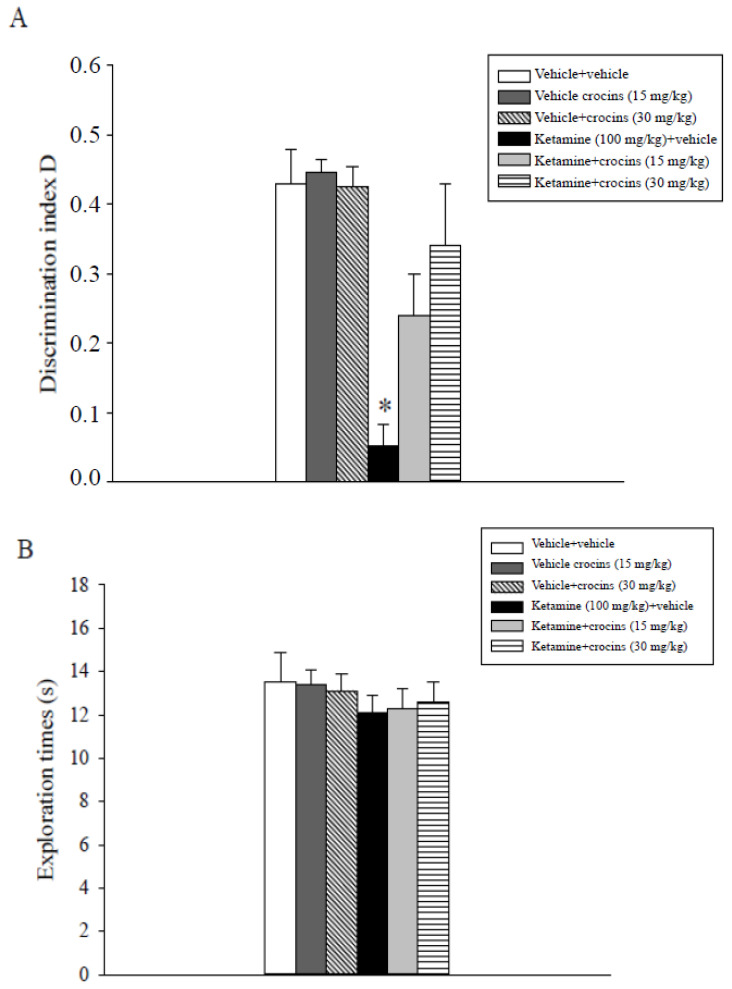
Object location task. The histogram represents the mean ± S.E.M. of 8 rats per treatment group. (**A**) Discrimination index D performance expressed by different groups of rats during T2. * *p* < 0.05 vs. all the other groups including the ketamine + crocins (15 mg/kg) and ketamine + crocins (30 mg/kg) groups. (**B**) Total exploration times.

Discrimination index D index data analysis showed a statistically significant main effect of ketamine (F(1,47) = 27.99, *p* < 0.001), a statistically significant main effect of crocins (F(2,47) = 4.06, *p* = 0.024) and a significant interaction between ketamine and crocins (F(2,47) = 4.09, *p* = 0.024). Subsequent post hoc comparisons indicated that the ketamine- and vehicle-treated rats displayed a significantly inferior index D with respect to all the other populations of rats, including the ketamine + 15 mg/kg crocins and ketamine + 30 mg/kg crocins groups (*p* < 0.05; Figure 2A).

Assessment of the exploratory activity data obtained for the various groups of rats during the choice phase (T2) did not show significant effects of ketamine, crocins or their combination (Figure 2B). The results indicate that crocins counteracted anesthetic ketamine-induced spatial recognition memory deficits.

### 2.3. Experiment 3: Effects of Acute Challenge with Crocins and Anesthetic Ketamine on Animals’ Performance in the HT

**Figure 3:**
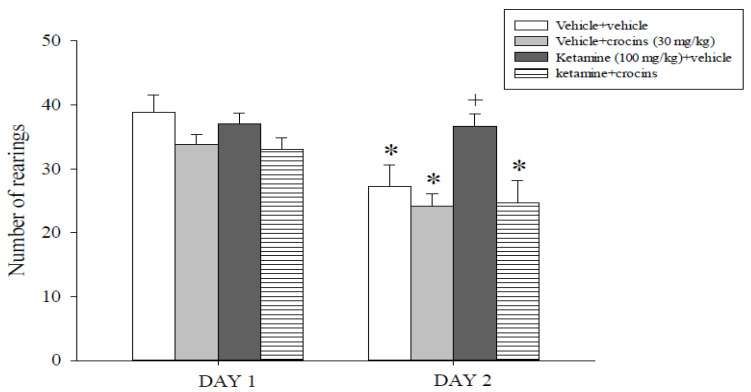
Habituation test. The histogram represents the mean ± S.E.M. of 8 rats per treatment group. * *p* < 0.05 vs. the same groups of rats on Day 1; ^+^ *p* < 0.05 vs. all the other groups on Day 2.

The HT results are reported in Figure 3. Overall analysis of the number of rearings revealed an effect of crocins (F(1,63) = 12.3, *p* < 0.001) and of trials (F(1,63) = 18.8, *p* < 0.001) but not an effect of ketamine or an interaction between ketamine, crocins and trials. A post hoc analysis carried out on these data indicated that the vehicle + vehicle, vehicle + crocins and ketamine + crocins groups but not the ketamine + vehicle group expressed significantly lower numbers of rearing episodes during Day 2 with respect to their performance during Day 1 (*p* < 0.05).

A two-way ANOVA test conducted on results related to rats’ performance during Day 1 did not evidence any effect either of ketamine or of crocins or an interaction ketamine x crocins. Conversely, evaluation of the Day 2 data showed a main effect of crocins (F(1,31) = 5.9, *p* = 0.022) but not an effect of ketamine or an interaction between ketamine and crocins. Post hoc comparisons showed that the ketamine + vehicle group displayed a significantly higher number of rearings compared to all the other experimental groups, including the ketamine + 15 mg/kg crocins and ketamine + 30 mg/kg crocins groups (*p* < 0.05).

The high number of rearing episodes displayed by the ketamine + vehicle-treated rats is an indication of an impairing effect caused by anesthetic ketamine on the associative learning abilities of the rats. The reduction of it by crocins suggests that this bioactive component of saffron counteracted the disruption of associative memory induced by anesthetic ketamine.

## 3. Discussion

According to previous reports, anesthetic ketamine impaired rodents’ performance in either the ORT [6,9,29,30] or OLT [6], two procedures reflecting non-spatial and spatial recognition memory, respectively. Interestingly, for the first time to our knowledge, anesthetic ketamine also disrupted the performance of animals in the HT, a behavioral paradigm assessing associative learning [28].

An acute challenge with crocins (15–30 mg/kg) counteracted the impairing effects caused by anesthetic ketamine on cognition evidenced in the above-reported behavioral paradigms. A per se effect of crocins was not revealed. In both recognition memory studies (ORT and OLT) and in the associative learning experiment (HT), retention was assessed 24 h after the training trial. This means that the effects of the compounds were revealed in procedures assessing long-term memory. Concerning the ORT and OLT, the chemicals were injected immediately after the sample phase. This suggests that ketamine and crocins might have acted on post-training memory components (the storage and/or retrieval of information). Conversely, in the habituation test, the compounds were administered 24 h before the training trial. The latter implies that the compounds could critically have exerted their effects on the encoding of information, although possible effects on the consolidation and retrieval of information can also be considered.

The present results provide new information with respect to previous findings of ours in which crocins were found to be able to attenuate the non-spatial recognition memory deficits produced by sub-anesthetic ketamine [21]. Specifically, in the present experiments, crocins counteracted anesthetic ketamine’s amnestic effects in different behavioral paradigms, reflecting non-spatial recognition but also spatial recognition and associative memory, respectively. Moreover, the current findings suggest that crocins modulated the memory disturbances induced by anesthetic ketamine in procedures evaluating not only the storage and retrieval but also the acquisition of information.

The compounds were administered peripherally. It cannot be ruled out, therefore, that unspecific factors (e.g., sensorimotor or motivational) might have influenced rats’ performance in the various memory tasks. It has been shown, however, that the exploratory levels displayed by animals either in the ORT and OLT during the retention trial (Day 2) and the number of rearing episodes expressed by rats during the training trial (Day 1) in the HT were not different among the various experimental groups. This pattern of results, thus, suggests that the implication of non-specific factors in the effects of anesthetic ketamine and crocins on animals’ cognitive performance can probably be excluded.

The mechanism(s) underlying anesthetic ketamine’s adverse effects, has (have) been, at least partially, attributed to its inhibitory effect on the NMDA receptor. It has been demonstrated that anesthetic ketamine inhibits glutamatergic neurotransmission by reducing glutamate release and suppressing axonal conduction, polysynaptic potential and cell excitability [31,32]. Anesthetic ketamine seems to reduce NMDA-mediated responses by two distinct mechanisms: (a) anesthetic concentrations of ketamine seem to block the open channel of the NMDA receptor and thereby decrease the mean channel-open time, and (b) reducing the frequency of channel opening through an allosteric mechanism [33]. A recent report has indicated that cognitive impairments induced by anesthetic ketamine might be dependent on its suppressing effects on astrocyte-mediated slow inward current (SIC) synchronization [34].

There is also evidence that alternative target sites, such as the GABA_A_ and the nicotinic cholinergic receptor, might be implicated in the amnestic effects of anesthetic ketamine. In this context, it has been reported that the potential interaction of ketamine with the GABA_A_-benzodiazepine receptor [29] and the α7 sub-unit of the nicotinic acetylcholine receptor [30] might also be involved in anesthetic ketamine’s amnestic action.

The mechanism(s) through which crocins reversed anesthetic ketamine’s impairing action on memory is (are) not yet elucidated and is (are) still a matter of investigation. In this context, it has been shown that saffron extracts and crocetin (the hydrolysis product of crocins) but not crocins partly antagonize the NMDA receptor by binding to the PCP binding site of it. In addition, both chemicals display an affinity for the sigma (σ) 1 receptor [35]. This apparent failure of crocins to bind at the NMDA receptor might depend on pharmacokinetic issues and, in particular, their poor intestinal absorption after oral administration in the rat [35].

Another study confirmed the above-mentioned antagonistic effects of saffron and crocetin on the NMDA receptor since both molecules reduced glutamatergic transmission in rat cortical brain slices [36]. Importantly, this partial blocking of the NMDA receptor might be critical for the presumed therapeutic effects of saffron and its bioactive constituents [35].

Furthermore, it has been reported that anesthetic ketamine interferes with the ERK signal transduction pathway since it has been shown that the inhibition of ERK by anesthetic ketamine underlies the memory impairments observed in the young rat [12]. Interestingly, it has recently been shown that crocins were able to reverse memory deficits caused by a challenge with hyoscine and normalized ERK levels in the rat hippocampus [37].

Anesthetic ketamine was also found to induce the overexpression of the transcription marker c-fos, which is an index of neuronal damage [13]. By contrast, in a series of studies, the beneficial action of saffron and crocins against cognitive dysfunctions, a typical feature of neurodegenerative diseases such as Alzheimer’s disease, or cerebral ischemia has been revealed [38]. In particular, crocins (injected intraperitoneally) were found to exert an inhibitory action on acetylcholinesterase activity [39] and demonstrated efficacy in counteracting β-amyloid (Αβ)-induced cognitive deficits [40] and c-fos overexpression in rats [41]. In this context, it has been revealed that the oral administration of crocins conferred neuroprotection in a rat model of cerebral ischemia [42].

A correlation between anesthetic ketamine and oxidative stress has also been proposed. It has been demonstrated that anesthetic ketamine impaired mitochondrial function and potentiated superoxide dismutase activity in the rat brain [14]. Regarding this latter issue, the well-known anti-oxidant properties of crocins may provide an alternative explanation for the findings reported here. Specifically, the oral or intraperitoneal administration of crocins attenuated memory deficits caused either by streptozotocin or chronic stress and enhanced anti-oxidant activity in rodents [22,23].

Collectively, it can be hypothesized that the partial antagonistic affinity of saffron and crocetin for the NMDA receptor, and the beneficial role played by crocins in the ERK pathway and c-fos expression along with their potent anti-oxidant properties might be critical for counteracting the amnestic effects of anesthetic ketamine.

The present work has some limitations. The current results might be considered preliminary and are limited to behavioral findings. Further research (e.g., molecular, biochemical and electrophysiological studies) is required to elucidate the mechanism(s) of action underlying crocins’ anti-amnestic effects.

In summary, the present results show, for the first time to our knowledge, that a bioactive constituent of saffron (crocins) exerts a modulatory role on anesthetic ketamine’s amnestic effects. The findings reported here might be of importance since ketamine is largely utilized in clinical and veterinary medicine in anesthesia and perioperative analgesia.

## 4. Materials and Methods

### 4.1. Animals

Different populations of male (3-month-old) Wistar rats (Hellenic Pasteur Institute, Athens, Greece) weighing 250–300 g were used for each experiment. The rats were housed in Makrolon cages (47.5 cm length × 20.5 cm height × 27 cm width), with three per cage, in a standard environment (21 ± 1 °C; 50–55% relative humidity; 12 h/12 h light/dark cycle, lights on at 7 a.m.) with access to food and water ad libitum.

The experiments that involved animals and their care were conducted in accordance with international guidelines and national (Animal Act, P.D. 160/91) and international laws and policies (EEC Council Directive 86/609, JL 358, 1, 12 December 1987). The present study was approved by the local committee (Prefecture of Larissa, Greece, protocol number 255200/1 October, 2020).

### 4.2. Behavior

#### 4.2.1. Object Recognition Task (ORT)

The ORT assesses non-spatial recognition memory abilities in rodents. This paradigm lacks a reward, and it is based on the spontaneous exploratory behavior of rodents [26]. The test apparatus consisted of a dark open box made of Plexiglas (80 cm length × 50 cm height × 60 cm width) that was illuminated by a 60 W light suspended 60 cm above the box. The light intensity was equal in the different parts of the apparatus. The objects to be discriminated (in triplicate) were made of glass, plastic or metal, and were in three different shapes—metallic cubes, glass pyramids and plastic cylinders 7 cm high—and could not be displaced by rats.

The ORT was performed as described previously [6]. Briefly, during the week before undertaking the testing, the animals were handled twice a day for 3 consecutive days. Before testing, the rats were allowed to explore the empty apparatus for 2 min for 3 consecutive days. During testing, a session that consisted of two trials was conducted. During the “sample” trial (T1), two identical samples (objects) were positioned in two opposite corners of the apparatus in a casual fashion, 10 cm away from the sidewalls. A rat was gently positioned in the center of the arena and allowed to inspect the two similar objects. After the sample phase (T1), the rat went back to its home cage, and an intertrial interval (ITI) followed. Subsequently, the “choice” trial (T2) was conducted. During T2, a novel object substituted one of the objects presented during T1. The animals, thus, were re-exposed to two objects: a copy of the familiar (F) object and the novel (N) object. All the combinations and positions of the objects were counterbalanced to reduce the potential bias due to preferences for specific places or objects.

Directing the nose toward the object at a distance no more of 2 cm and/or touching the object with the nose was considered exploratory behavior. Turning around or sitting on the object was not considered exploratory behavior. The total time spent by the rats exploring the two identical objects (F1 and F2) during the sample phase (T1) and the total time spent exploring the two different objects (F and N) during the choice trial (T2) were manually recorded by using a stopwatch. The discrimination between F and N during T2 was measured by comparing the time spent exploring the familiar object with the time spent exploring the novel object. Because the exploratory time may be influenced by differences in the total exploratory activity, a discrimination index (D) representing the preference for the new as opposed to familiar object was calculated as follows: D = N − F/N + F, where N is the exploration time for the novel object, F is that for the familiar object and N+F is the total exploration time for both objects during T2 [43]. Correct recognition was shown by rats consistently spending more time inspecting a novel object than the familiar one during T2 [26].

#### 4.2.2. Object Location Task (OLT)

The OLT is a version of the ORT that evaluates spatial recognition memory. This task assesses the ability of rodents to discriminate the novelty of the object locations but not the objects themselves because the testing arena is already familiar to the animals [27]. The testing arena was that utilized in the ORT. The apparatus was placed in a large observation room and was surrounded with external large and typical objects (cues) to assist the animals to successfully perform the test. These cues were kept in a fixed position for the entire testing period. The objects were the same objects as in the ORT.

The OLT was performed as described elsewhere [27,44]. Briefly, during the week before undertaking testing, the animals were handled twice daily for 3 consecutive days. Before testing, the rats were allowed to explore the empty apparatus for 2 min for 3 consecutive days. During testing, a session that consisted of two trials was carried out. During the “sample” trial (T1), two identical samples (objects) were positioned in two opposite corners of the apparatus in a casual fashion, 10 cm away from the sidewalls. A rat was gently positioned in the center of the arena and allowed to inspect the two similar objects. After the sample phase (T1), the rat went back to its home cage, and an intertrial interval (ITI) followed. Subsequently, the “choice” trial (T2) was conducted. During T2, one of the two similar objects was relocated to a different position (new location (NL)), while the other object remained in the same position (familiar location (FL)) as in the sample phase (T1). Thus, the two objects were now in diagonal corners. All the combinations and positions of the objects were counterbalanced to reduce the potential bias due to preferences for specific places or objects.

The definition of exploration is described above in the object recognition protocol. The time spent by the rats exploring each object during T1 and T2 was manually recorded with a stopwatch. The discrimination between the FL and NL during T2 was measured by comparing the time spent exploring the object in the FL with the time spent exploring the object in the NL. Because the exploratory time may be influenced by differences in the total exploratory activity, a discrimination index (D) representing the preference for the new as opposed to familiar object position was calculated as follows: D = NL − FL/NL + FL, where NL is the exploration time for the object in the novel position, FL is that for the object in the familiar location and NL + FL is the total exploration time for both objects during T2 [43]. Correct recognition is shown by rats spending consistently more time inspecting the novel place of the object than the familiar one during the choice trial T2 [26].

#### 4.2.3. Habituation Test (HT)

The retention of a habituation to a novel environment reflects a non-associative, non-aversive form of learning. It can be quantified by the number of rearing episodes expressed by rodents in each test trial. A decrement in rearing episodes during the retention trial is considered to be an index of the intact non-associative memory abilities of rodents [28]. The apparatus consisted of a box made of Plexiglas (41 cm length × 33 cm height × 41 cm width). The test was performed as described previously [28]. Each animal was placed into the test arena, and the number of rearing episodes was recorded for 5 min. During testing, a session that consisted of two 5 min trials was performed. An intertrial period of 24 h was utilized.

In all the above-reported behavioral procedures, in order to avoid the presence of olfactory traces, devices and objects (where necessary) were washed with a solution containing 20% alcohol following each trial and then dried with sanitary towels.

### 4.3. Drugs

The crocins used in the current experiments were derived from the same batch of plant material (saffron) and the same purification procedure, extraction and separation. Our plant material was kindly offered by the Cooperative of Saffron, Krokos, Kozani, Greece.

**Figure 4:**
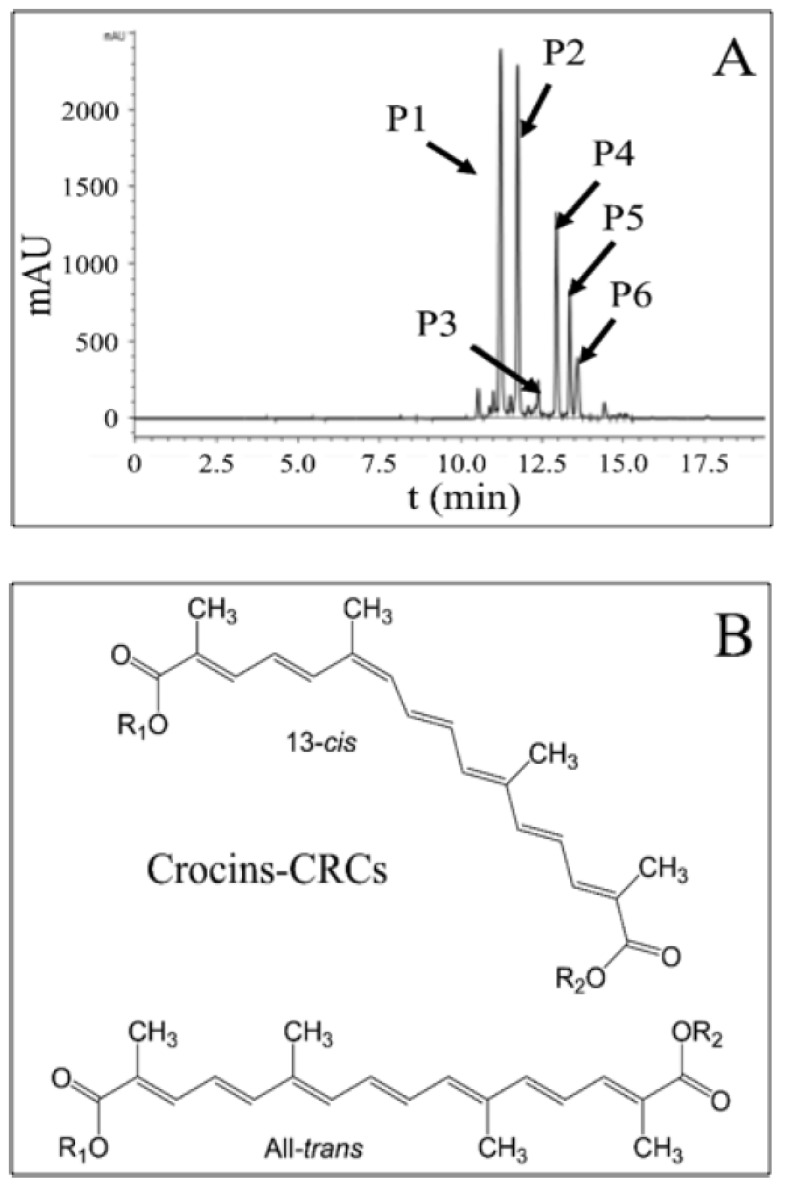
Chromatogram of crocin extract at 440 nm and structures of crocins. Identification of crocins according to HPLC analysis was performed, comparing the UV-vis spectra and the retention times (tR) of the peaks with literature data and MS data. The identified peaks presented are as follows: P1: trans-4GG, P2: trans 3Gg, P3: trans 2gg, P4: cis4 GG, P5: trans 2G, P6: cis crocin (**A**). The most abundant were trans-crocin 4 and trans-crocin 3. Nomenclature of crocins was based on the proposal of Carmona et al., i.e., the first part describes the cis/trans form of the aglycon part, followed by the total number of sugar moieties (glycose monomers), and finally, the type of sugar in each part of the crocin structure is indicated. Namely, G refers to gentiobiose and g, to glucose (2) (**B**).

The crocins were isolated from the red dried stigmas of *C. sativus* using a slightly modified method described previously [45]. They were purified from the stigmas after successive and exhaustive extraction with (a) petroleum ether at 40–60 °C, (b) diethyl ether (Et_2_O) and (c) 80% methanol (MeOH) using ultrasound-assisted extraction. The ultrasound extraction was performed in a Sonorex, Super RK 255H type (300 × 150 × 150 mm internal dimensions) ultrasound water bath (indirect sonication), at a fixed frequency of 35 kHz. The temperature of the sonicated water was 25 °C. Procedures (a) and (b) took place in order for the stigmas to be free of the picrocrocin and safranal, respectively. The methanol extract, after evaporation (condensed to dryness) under vacuum at room temperature, provided crocins, which were dark red powder residue. Crocins are unusual water-soluble carotenoids (glucosyl esters of crocetin). The major component is a digentiobiosyl ester of crocetin (approximately 60% of the total crocins) [46]. The chemical profile of crocins has been well documented in previous studies [14,47,48,49], and we evaluated the quality of the fresh prepared extract used in this study. The purity of the crocins was 85% (according to HPLC., Figure 4).

The crocins were dissolved in saline (NaCl 0.9%) and were administered intraperitoneally (i.p.). The doses of crocins (15 and 30 mg/kg) were selected based on previous findings [21].

Ketamine hydrochloride (Sigma, St Louis, MO, USA) was dissolved in saline and administered i.p. at the anesthetic dose of 100 mg/kg [6].

All drug solutions were freshly prepared on the day of testing and were administered i.p. in a volume of 1 mL/kg. For all studies, control animals received isovolumetric amounts of the specific vehicle solutions.

### 4.4. Experimental Protocol

**Figure 5:**
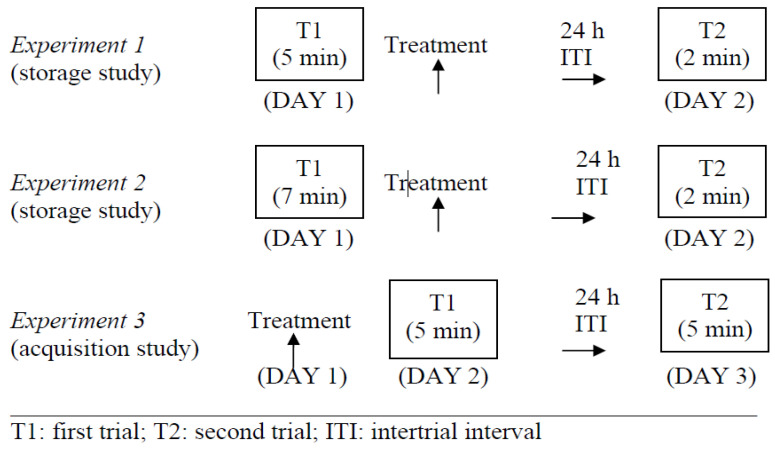
Summary of the experimental protocol.

Given the fact that the hypothermic properties of ketamine exert a protective effect on rats’ recognition memory [6], the different groups of animals received treatment in a “warm” (25 °C) room to avoid hypothermia and remained there under these conditions for 2 h, starting immediately after injection [6,9,29,30]. Testing was carried out under standard environmental conditions (21 °C) 24 h after treatment, when a complete recovery of animals’ sensorimotor functions was achieved [6,7,8,9,29,30]. The experimental protocol is summarized in Figure 5.

An *anesthetic state* was defined as the loss of the righting reflex and movement. The animals’ behavior was video recorded. Data evaluation was subsequently performed by an experimenter who was unaware of the pharmacological treatment of each subject.

#### 4.4.1. Experiment 1: Effects of Acute Challenge with Crocins and Anesthetic Ketamine on Animals’ Performance in the ORT

Animals were randomly divided into six experimental groups with 8 rats per group as follows: vehicle + vehicle; vehicle + crocins (15 mg/kg); vehicle + crocins (30 mg/kg); vehicle + ketamine (100 mg/kg); ketamine (100 mg/kg) + crocins (15 mg/kg); ketamine (100 mg/kg) + crocins (30 mg/kg). Appropriate treatment was performed immediately after the training (sample) trial T1. The crocins were administered 5–10 s after the vehicle or ketamine.

In this study, the duration of the sample trial T1 was increased from 2 to 5 min, since under this condition, it has been shown that control rats’ non-spatial recognition memory abilities can be preserved for 24 h, while the duration of the choice phase remained unchanged (2 min) [29,30].

#### 4.4.2. Experiment 2: Effects of Acute Challenge with Crocins and Anesthetic Ketamine on Animals’ Performance in the OLT

Animals were randomly divided into six experimental groups with 8 rats per group as follows: vehicle + vehicle; vehicle + crocins (15 mg/kg); vehicle + crocins (30 mg/kg); vehicle + ketamine (100 mg/kg); ketamine (100 mg/kg) + crocins (15 mg/kg); ketamine (100 mg/kg) + crocins (30 mg/kg). Appropriate treatment was performed immediately after the training (sample) trial T1. The crocins were administered 5–10 s after the vehicle or ketamine.

In this study, the duration of the sample trial T1 was increased from 2 to 7 min, since under this condition, it has been shown that control rats’ spatial recognition memory abilities can be preserved for 24 h, while the duration of the choice phase remained unchanged (2 min) (our unpublished observations).

#### 4.4.3. Experiment 3: Effects of Acute Challenge with Crocins and Anesthetic Ketamine on Animals’ Performance in the HT

Animals were randomly divided into four experimental groups with 8 rats per group as follows: vehicle + vehicle; vehicle + crocins (30 mg/kg); vehicle + ketamine (100 mg/kg); ketamine (100 mg/kg) + crocins (30 mg/kg). Appropriate treatment was performed 24 h before starting the training (sample) trial T1. The crocins were administered 5–10 s after the vehicle or ketamine.

### 4.5. Statistical Analysis

Data are expressed as mean ± S.E.M. The data of Experiments 1 and 2 were analyzed using two-way analysis of variance (ANOVA). The factors were crocins and ketamine. Experiment 3’s data were analyzed using three-way ANOVA with the between-subjects factors crocins and ketamine and the within-subjects factor trials. Further analyses were carried out separately for each trial (day) using the two-way ANOVA test. The factors were crocins and ketamine. Post hoc comparisons between the treatment means were made using Tukey’s t-test. Values of *p* < 0.05 were considered statistically significant [50].

## Data Availability

The data presented in this study are available on request from the corresponding author.

## Sample Availability

Samples of crocins and ketamine are available from the authors.
